# Neuropathic Pain in Cancer: What Are the Current Guidelines?

**DOI:** 10.1007/s11864-024-01248-7

**Published:** 2024-08-05

**Authors:** Matthew R. Mulvey, Carole A. Paley, Anna Schuberth, Natalie King, Andy Page, Karen Neoh

**Affiliations:** 1 Leeds Institute of Health Sciences, School of Medicine, Faculty of Medicine and Health, University of Leeds Level, 10 Worsley Building, Clarendon Road, Leeds, LS2 9LN UK; 2 Academic Unit of Palliative Care, St Gemma’s Hospice, Leeds, UK

**Keywords:** Neuropathic cancer pain, Guidelines, Chronic pain, Treatment, Management

## Abstract

Neuropathic cancer pain is experienced by 30–40% of patients with cancer. It significantly reduces quality of life and overall wellbeing for patients living with and beyond cancer. The underlying mechanisms of neuropathic pain in patients with cancer are complex and involve direct tumour involvement, nerve compression or infiltration, chemotherapy and/or radiotherapy-induced nerve damage, or post-surgical complications. It is crucial for healthcare professionals to assess and manage neuropathic cancer pain effectively. There is increasing recognition that standardisation of neuropathic pain assessment leads to tailored management and improved patient outcomes. Pain management strategies, including medication, interventional analgesia, physical and complementary therapy, can help alleviate neuropathic pain and improve the patient's comfort and quality of life.

## Introduction

Pain is one of the most common symptoms reported by cancer patients. It can originate from visceral, bone or nerve tissue and can have acute and/or inflammatory nociceptive mechanisms, as well as neuropathic mechanisms, including nociplastic (central sensitisation) involvement [1, 2]. Pain in cancer patients is often a mixed-pain syndrome, rarely presenting as purely nociceptive or neuropathic [3]. The prevalence of pain in patients with cancer varies according to tumour site, stage of disease, exposure to anti-cancer therapies and setting of care. Moderate to severe cancer pain affects 40–60% of adult cancer patients [4, 5], 64% of patients with advanced disease report pain [5], and 40% of patients with cancer are affected by neuropathic cancer pain (NCP) [6]. At least a third of patients with cancer pain are undertreated [7, 8], and this is often associated with inadequate attention and assessment of pain during routine oncology contacts [9].

The aetiology of NCP is complex and includes direct nerve invasion or compressions by solid tumours, as well as neural toxicity, chemotherapy and radiotherapy [10, 11]. NCP severity and temporal characteristics vary over time; it can be acute or chronic with continuous or episodic features [12]. NCP is challenging to manage clinically as it often co-occurs with nociceptive and inflammatory pain mechanisms [13] and commonly requires adjuvant antidepressant or anticonvulsant drugs [14]. Large population survey data indicate that NCP is associated with significantly higher levels of depression, chronicity, adjuvant analgesic use and breakthrough pain, as well as lower quality of life [8, 13]. Persistent uncontrolled NCP is likely to become physically and emotionally disabling, resulting in increased suffering and reduced quality of life.

The clinical characteristics and management of NCP differ from purely nociceptive and inflammatory cancer pain. It is important to differentiate NCP from other types of cancer pain, as it is associated with worse pain outcomes and requires different treatment strategies [15]. Routine assessment of pain in patients with cancer, using standardised tools, is necessary to identify underlying aetiology and mechanisms, and to guide treatment [1].

At the bedside, neuropathic pain is characterised by the positive or negative of somatosensory phenomena; that is to say, either gain or loss of sensory function expressed as hypersensitivity or numbness, which can often co-occur in the same or adjacent dermatomes [16].

Screening tools designed to identify neuropathic pain signs and symptoms, in populations of patients with chronic pain, have been widely reported [17]. The Leeds Assessment of Neuropathic Symptoms and Signs (LANSS) [18], Douleur Neuropathique en 4 (DN4) [19] and painDETECT (PDQ) [20] have all demonstrated acceptable discriminatory performance in patients with neuropathic versus non-neuropathic cancer pain [21]. However, these screening tools were found to be less sensitive in patients with tumour-related neuropathic pain compared with treatment-related neuropathic pain [21]. Nevertheless, until the widespread adoption of standardised diagnostic algorithm for neuropathic cancer pain (see Shkodra et al. [13]), screening tools offer a practical approach to identify cases of predominately neuropathic cancer pain.

There is robust evidence for the use of strong opioids for the management of cancer pain [22]; however, there is a lack of good quality clinical trial data on the use of analgesic medication for NCP [14]. This is due, in part, to the complex and heterogeneous nature of NCP. As discussed by Shkodra & Caraceni [14], the causal mechanisms of NCP are varied and not fully understood; consequently, defining a homogeneous study population for clinical trials of novel analgesic therapies is extremely challenging. While the underlying mechanisms of NCP are so varied, the mechanism of action of analgesic therapies are often very specific. It is therefore unsurprising that there are so few effective pharmacological agents. The application of standardised NCP diagnostic criteria and phenotyping patients into mechanistic subgroups (using quantitative sensory testing) [23], will increase the homogeneity of study participants in clinical trials and, in turn, the likelihood of identifying effective analgesic agents.

## Pharmacological Treatment

### Basic Principals

Fundamental to the management of NCP is standardised pain assessment that occurs at every engagement between a patient with cancer and a healthcare professional [24, 25]. As a minimum, cancer pain assessment should establish the type of cancer pain (based on the ICD-11 taxonomy for chronic cancer pain [10]), site of origin, underlying mechanisms (nociceptive (somatic/visceral), neuropathic, mixed), intensity, and impact on sleep, daily activities, and quality of life [21, 26]. The heterogenous nature of NCP means accurate diagnosis at the bedside is challenging, and there is no internationally accepted gold standard for NCP assessment.

A grading system for neuropathic pain was published in 2008 by the Neuropathic Pain Special Interest Group (NeuPSIG) of the International Association for the Study of Pain (IASP) [27]. This grading system has been revised and adapted for patients with cancer-related pain, but still requires validation in clinical populations [16, 28].

The WHO analgesic ladder has been the central idea of cancer pain relief for over three decades. [29]. However, the analgesic ladder it has been criticised for its “one size fits all” approach which does not differentiate between nociceptive and neuropathic pain, for which there are distinct management pathways [14, 25]. The WHO cancer pain guidance was updated in 2018 [30] with less focus on the ladder and more on individualised therapeutic planning based on careful assessment of each individual patients’ pain [30]. The 2018 WHO guidance recommends that the type and strength of analgesic is determined by the type and severity of pain [30]. This allows for the management of NCP with mono or combination therapy “depending on clinical assessment and pain severity in order to achieve rapid effective and safe pain control” [30]. The 2018 WHO guidance is primarily focused on ‘opioid management’ of ‘cancer pain’; there is a lack of high-quality randomised control trial evidence to support clinical practice in the use of opioids for NCP [31, 32]. In practice, the complex pathophysiology of NCP often requires concurrent use of opioids and neuropathic adjuvants to achieve effective pain management [14, 33].

### Weak Opioids

Weak opioids (i.e., codeine, tramadol) are used as monotherapy or in combination with adjuvant analgesics for managing moderate pain. Both codeine and tramadol are primarily μ-opioid receptor agonist [34]; although tramadol has monoaminergic properties that may contribute to its analgesic effect in the treatment of neuropathic pain [35]. Clinical trial data indicate equivalent analgesia between tramadol and codeine-plus-paracetamol for moderate cancer pain (including NCP) [36], and for cancer patients with neuropathic pain (not specifically cancer-related neuropathic pain) [37]. Both studies reported higher adverse events associated with tramadol.

### Limitations of Weak Opioids in the Management of NCP

Weak opioids have a therapeutic ceiling effect which has led to debate about their usefulness in treating moderate cancer pain (including moderate intensity NCP), in favour of early initiation of strong opioids at low doses [22, 38]. According to the European Association for Palliative Care (EAPC), low doses of strong opioids should be considered in place of weak opioids for managing moderate cancer pain [35]. Low-dose strong opioids are potentially more appropriate for managing NCP, as one of its defining characteristics is higher pain intensity compared with non-neuropathic cancer pain [6].

Initiating analgesic treatment of NCP with low dose strong opioids may achieve equivalent pain relief with fewer side effects, and may be more efficient, i.e. achieve pain relief quicker than weak opioids [33]. Moreover, in countries where opioids are limited, for example in low- and middle-income countries, weak opioids are often more expensive than strong opioids, so managing moderate cancer pain with low-dose strong opioids increases access to effective pain management [22].

### Strong Opioids

There is a lack of good quality, randomised clinical trial data on the use of strong opioids for NCP [14]. According to a number of European guidelines, there are no major differences between morphine, oxycodone, and hydromorphone administered via the oral route, and any can be considered in the first instance for managing moderate to severe cancer pain, including NCP [14, 32, 33, 35]. Although opioids can be used alone to manage NCP, the response is often low, requiring higher doses, [39]. For example, in one clinical trial of 57 patients with non-cancer related neuropathic pain, the mean maximum daily dose was 45.3 ± 3.9 mg/day [40]. In contrast Dou et al. [41] reported the minimum effective daily-dose of morphine monotherapy was 247.5 ± 80.0 mg/day (approximately 5.5 times higher) in 40 patients with NPC.

#### Morphine

Although morphine provides the cornerstone of pharmacological treatment of cancer pain [29, 33, 35, 42], good quality clinical trial data are lacking for NCP. In a small study of 40 cancer patients with severe neuropathic pain [41], the minimum effective daily-doses of morphine monotherapy was 247.5 ± 80.0 mg/day; this reduced to 184 ± 69.9 mg/day when combined with pregabalin. A 2016 Cochrane Review of oral morphine for cancer pain [43] identified one (low quality) study reporting data on 40 cancer patients, in which “morphine was superior in neuropathic pain” [44].

#### Oxycodone

Oxycodone is a semi-synthetic μ-opioid receptor agonist [45, 46]. A 2022 Cochrane review found no difference between oxycodone taken every 4–6 h (immediate-release) or every 12 h (controlled-release) for non-specific cancer pain [46]. In addition, no difference was found between oxycodone and other strong pain killers such as morphine; however, less frequent somnolence, hallucinations and muscle twitching were observed for oxycodone [46]. A Spanish prospective epidemiological study of neuropathic pain in cancer patients reported 50% achieved adequate pain relief after 1 month of oxycodone compared to 40.8% receiving “other opioids” and 37.3% receiving no opioids [47].

#### Tapentadol

Tapentadol is a μ-opioid receptor and selective noradrenalin reuptake inhibitor within the central nervous system (CNS) and has been suggested for NCP [14]. It has similar chemical structure to tramadol, with stronger analgesic effect; however, the analgesic strength of tapentadol is approximately three times lower than that of morphine (when administered via the oral route) [48]. The place of tapentadol as a weak or strong opioid is debated. In the UK tapentadol is considered a strong opioid for managing moderate to severe acute or chronic pain in adults [49] However, elsewhere Tapentadol is considered a weak opioid when used to manage chronic pain in cancer patients [50] Takemura et al. [51] reported data on 29 cancer patients with neuropathic pain who received tapentadol monotherapy. The authors reported minimal effective daily dose of tapentadol of 30.8 ± 42.0 mg/day [51].

#### Fentanyl

Fentanyl is a synthetic μ-opioid agonist, and has 100 times the analgesic strength of morphine. Its lipophilic properties mean it is ideally delivered via a transdermal patch which requires replacement every 72 h [48]. Due to the time taken to titrate analgesia, transdermal opioids are most appropriate for patients with relatively stable pain [14, 32, 33, 35]. Analgesic effect takes between 12–24 h, necessitating effective alternative rescue analgesia during this period. During transdermal fentanyl therapy, breakthrough pain is usually treated using immediate release oral morphine or oxycodone [48].

In a sample of 26 cancer patients with neuropathic pain randomised to receive fentanyl monotherapy, Takemura et al. [51] reported minimal effective daily dose of 21.9 ± 28.7 mg/day. Haumann et al. [52] randomised 26 cancer patients with a neuropathic pain component (defined using the Douleur Neuropathique 4 Questionnaire (DN4 screening tool [19]) to receive fentanyl monotherapy of whom 15% achieved at least 50% reduction in pain after 1 week of treatment. When compared with sustained-release morphine formulations in an open label trial, transdermal fentanyl has been shown to be as effective in the treatment of non-specific cancer pain and has 30% lower incidence of adverse effects such as constipation and sedation [53].

#### Buprenorphine

Buprenorphine is a partial agonist of μ-opioid receptors and agonist of ĸ-opioid receptors. It has approximately 75 times the analgesic strength of morphine [48]. Like fentanyl, buprenorphine is delivered via transdermal patch applied on the skin every 72–96 h; although it is available in oral and sublingual formations as well [48]. RCT evidence of effect of buprenorphine for NCP is lacking [54].

#### Methadone

Methadone is a synthetic agonist of μ- and δ-opioid receptors, as well as an antagonist of NMDA receptor [55]. Given the mixed mechanism of action at both opioid and NMDA receptors, it has been suggested that methadone may have an important role to play in patients with NCP [52, 55]. However, methadone presents some challenges in dose titration and is recognised to be not infrequently associated with adverse effects including potentially fatal arrhythmias in some patients [55]. Methadone should, therefore, be initiated by experience practitioners. Due to its specific pharmacokinetic characteristics and a long and unpredictable half-life, methadone requires careful and cautious individualisation of dosing schedules by experienced healthcare professionals when initiating or switching from high doses of other strong opioids [48, 52, 55, 56].

Takemura et al. [51] reported data from 32 cancer patients with neuropathic pain who received methadone monotherapy. The authors reported a mean minimum effective daily dose for methadone of 114.2 ± 104.6 mg/day. A 2017 Cochrane Review of methadone for non-specific cancer pain, reported data from six low-quality studies which indicate a similar analgesic effect when compared to morphine [55]. However, the authors noted that methadone should be considered for people who cannot tolerate other opioids. Haumann et al. [52] randomised 26 cancer patients with a neuropathic pain component (DN4 > 4) to receive methadone monotherapy of whom 50% achieved at least 50% reduction in pain after 1 week of treatment. However, large scale high quality clinical trials of methadone for NCP are lacking [56].

### Adjuvant Pharmacological Treatment

The complex pathophysiology of NCP often requires concurrent use of opioids and neuropathic adjuvants [14, 33]. Careful titration of neuropathic analgesic adjuvants is needed to prevent central nervous system issues and adverse effects; see later sections on anticonvulsants and antidepressants for details.

#### Anticonvulsants

Pregabalin and gabapentin are, with the antidepressants amitriptyline and duloxetine, first line adjuvant analgesics in patients with NCP [3, 32, 48, 57, 58, 59]. Concerns have been raised about the level of supporting evidence for the use of antidepressants and anticonvulsants for the treatment of neuropathic pain in patients with cancer [59, 60]. It has been noted that most guidelines were extrapolated results from publications in patients with a non-cancer diagnosis [59].

There may be a greater clinical benefit and effect on pain when gabapentinoids and a tricyclic antidepressant are co-administered [3]; however, this should be weighed against the potential for increased side effects [61]. In patients with NCP, the benefits of adjuvant analgesics are considered to outweigh the adverse effects [58]. The addition of gabapentin or pregabalin to an opioid as combination therapy can increase effectiveness [62] and may enable the dose of opioids to be reduced [63]. Given the limited evidence in cancer related neuropathic pain, if ineffective, adjuncts should be weaned and stopped [61].

A mild to moderate benefit of gabapentin for NCP is likely [39], although robust conclusions cannot be drawn due to the quality of evidence available. Some suggest that gabapentin achieves the best outcome for patients with neuropathic pain compared to non-neuropathic chronic musculoskeletal pain [64]. It is considered reasonable to trial gabapentin for NCP, including chemotherapy induced peripheral neuropathy, although evidence in inconclusive [65, 66].

Overall, there is limited good quality evidence for the benefit of pregabalin or gabapentin for managing NCP [67]. However, a small number of studies have shown a statistically significant reduction in cancer-related neuropathic pain when treated with pregabalin compared with placebo [68, 69].

Some guidelines suggest that carbamazepine, oxcarbazepine, sodium valproate and topiramate can be considered as third line options for NCP after opioids, antidepressants, and the antiepileptics gabapentin and pregabalin [48].

#### Antidepressants

Antidepressants are commonly used for NCP. Tricyclic antidepressants (TCA) and serotonin-noradrenaline reuptake inhibitors (SNRI) are most commonly used. The dose required for analgesia is usually lower than the antidepressant dose [31, 39]. However, TCAs have anticholinergic side effects (sedation, confusion, somnolence, dry mouth, bladder distension) and may be poorly tolerated, particularly in older patients [31]. SNRIs may be better tolerated compared with TCAs [11]. SNRIs such as duloxetine and venlafaxine have demonstrated effectiveness for chemotherapy induced peripheral neuropathy (CIPN) [11]. However, RCT data on the effect of TCAs and SNRIs on tumour-related neuropathic cancer pain are lacking.

Amitriptyline is considered a first line adjuvant analgesic for NCP [31, 32], and was the medication of first choice in several clinical practice guidelines [59]. Duloxetine is also suggested as a first line treatment for neuropathic pain, particularly if it is related to cancer treatment [70–72], including chemotherapy induced peripheral neuropathy [65]. Nortriptyline is an alternative antidepressant with similar efficacy to amitriptyline, and considered to have less toxicity, particularly cardiac side effects [11]. For patients with NCP, topical doxepin can be considered for the treatment of localised peripheral neuropathic pain [48], however this is based on limited evidence. Venlafaxine is not a first line adjuvant analgesic for NCP; however, it has shown some potential to prevent oxaliplatin induced peripheral neuropathy, which requires further investigation [65].

#### Cannabinoids

Medical cannabis, or cannabinoids, come in various combinations of tetrahydrocannabinol (THC) and cannabidiol (CBD) [73]. The evidence for their use in general cancer pain is unclear, and is lacking in NCP [74]. Fallon et al.[75] did not find superiority of Sativex (THC 27 mg.ml: CBD 25 mg/ml) compared to a placebo in reducing chronic pain from advanced cancer unalleviated by opioid therapy. Systematic review evidence has concluded that many studies of cannabinioids for caner pain (not specifically NCP) are under-powered and inconclusive [76, 77].

#### Ketamine

Ketamine is a derivative of phencyclidine [78] and has the benefit of not inducing significant respiratory depression. It is a dissociative anaesthetic which blocks MDNA receptors and is indicated when pain is unresponsive to opioids [78]. Some authors recommend its use as an analgesic alongside opioids as it reduces hypersensitivity at the dorsal horn [39]. However, the risk of serious adverse effects makes its use problematic and although small, under-powered studies show some benefit for cancer-related neuropathic pain, results are inconclusive and conflicting [31, 79].

## Interventional Procedures

### Interventional Analgesia

Spinal administration of local anaesthetics, opioids or clonidine should be considered for patients in whom analgesia is inadequate or who have intolerable adverse effects despite the optimal use of oral and parenteral opioids and non-opioid agents [35]. Other options such as the use of spinal cord stimulators may be appropriate for some patients with NCP, although analgesic effect may be limited compared to patients with non-malignant pain [80]. Suitability of spinal interventional analgesia should be based on anatomical distribution of the patient’s cancer, in addition to considering the neuroanatomical and dermatomal distribution of neuropathic pain [80].

### Radiotherapy

Cancer-related destruction of neural structures and bone can induce severe pain [81]. Cancer-related bone pain does not strictly result from a neuropathic injury, it is a distinct and complex pain state with neuropathic and inflammatory components [39]. Existing guidelines on the management of neuropathic pain reference the use of external beam radiotherapy in patients with localised cancer-related bone pain alongside the use of appropriate pharmacological and other management options [32, 48, 57, 64, 82].

### Ablation

Ablation techniques can be directed to act upon nerve structures (percutaneous neurolysis) assumed to be involved in neuropathic pain mediation, or directly upon the tumour itself (percutaneous ablation) to reduce inflammation and tumour compression of adjacent structures, including sensory nerves [83, 84]. A variety of ablation techniques can provide valuable neurolysis and tumour-directed pain palliative effects that can be incorporated within clinical guidelines for pain reduction in patients with NCP [83]. The most applied ablation techniques include cryoablation, radiofrequency, and microwave ablation [83, 84]. Ablative techniques generally involve needle probe insertion into the tumour or nervous structure, through which extreme hot/cold temperatures, radiofrequency currents or oscillating microwaves are passed causing cell death around the probe tip [83].

Radiofrequency ablation and cryoablation have both been applied for neurolysis [84]. Microwave ablation is rarely used for percutaneous neurolysis due to higher cost and lack of a specific thermocoagulation protocol [84]. Percutaneous neurolysis is usually performed under local anaesthesia and intravenous analgesia or conscious sedation since real time neurologic control is often helpful to ensure a safe and efficacious therapeutic session [83]. Imaging guidance and stimulation tests performed prior to thermocoagulation increase safety and efficacy of the technique. When compared with conservative pain therapy, percutaneous neurolysis can reduceopioid consumption and consequently long-term opioid-related side effects [83].

### Transcutaneous Electrical Nerve Stimulation (TENS)

Transcutaneous Electrical Nerve Stimulation (TENS) is inexpensive, non-invasive, often suitable for self-administration, and has no potential for toxicity or overdose [85]. A recent meta-analysis of 91 randomised control trials (n = 4841) of TENS for acute and chronic pain reported a significant overall effect in favour of TENS when compared to placebo; standardised mean difference of -0.96 (95% CI -1.14, -0.78) [85]. TENS has no drug interactions and is often used in combination with other analgesic medications. However, trial data are lacking for TENS or TENS-like devices in NCP management. A systematic review of treatments for chemotherapy induced peripheral neuropathy (CIPN) reported insufficient evidence support the use of acupuncture-like TENS for painful CIPN [70]. A Cochrane Systematic Review (2012) identified three RCTs of TENS for cancer pain in adults but results were inconclusive due to lack of robust trial data [86]. Bennett et al. (2010) conducted a feasibility trial of TENS for bone cancer pain and reported reduction in movement-related pain compared with placebo-TENS [87].

### Acupuncture

It has been theorised that acupuncture has the potential to activate descending pain control systems and inhibit pain-related ion channels and receptor activity [88, 89]. However, evidence supporting its use is weak and systematic reviews investigating the use of acupuncture in cancer are primarily concerned with chemotherapy-induced neuropathy, with inconclusive results [88]. Various systematic reviews, meta-analyses and narrative syntheses have found evidence to support the use of acupuncture for non-cancer-related neuropathic pain, but conclusions are weakened by heterogeneity, methodological shortcomings and low or very low-quality ratings [89].

### Challenges and Emerging Solutions for Managing NCP

A growing understanding of opioid-related adverse effects associated with chronic use, largely due to advances is long-term cancer survivorship, have driven advances in novel molecular research on opioid action. For example, research into mu opioid receptor function is providing insights into the variation in analgesic effectiveness and side effect profiles, offering novel targets for the development of new synthetic opioid analgesics [90].

Emerging data on the use of psycho-physical quantitative sensory testing (QST) as a biomarker for neuropathic pain suggests that phenotyping sensory function in patients with chronic cancer pain may help to identify sub-groups patients who are more likely to respond to treatments [23, 91].

The lack of standardised pain management guidelines remains a key barrier to effective NCP management and is associated with under-diagnosis and undertreatment. A large Korean observational study of 2003 cancer patients reported that 36% (95%CI 32.5–39.5) had NCP, of which less than half received the recommended neuropathic adjuvant analgesics [92]. However, clinical trial data show that implementing simple mechanism-based pain assessment into routine oncology practice increase access and tailoring of neuropathic analgesic [16, 93]. The implementation of standardised neuropathic cancer pain diagnostic algorithms, such as the EPAC/IASP NCP algorithm [23], into routine clinical practice is required.

## Summary

Effective management of NCP is challenging; the underlying mechanisms are complex, commonly with mixed pain pathophysiology and in patients with multi-morbidity. National and international cancer pain management guidelines indicate that opioids remain the cornerstone of NCP management [12, 30, 32, 33, 35, 38, 57, 59, 60]. Standardised treatment algorithms for the use of recommended adjuvant antidepressants, anticonvulsants and topical analgesics provide additional treatment options.

## Key References

- Taverner T. Neuropathic pain in people with cancer (part 2): pharmacological and non-pharmacological management. Int J Palliat Nurs. 2015; 21(8):380–4.
  - This reference is of importance because it describes, in detail, the pharmacological and nonpharmacological management of neuropathic pain in cancer that is accessible to clinical audiences.
- Reis-Pina P, Acharya A, Lawlor PG. Cancer pain with a neuropathic component: a cross-sectional study of its clinical characteristics, associated psychological distress, treatments, and predictors at referral to a cancer pain clinic. J Pain Symptom Manage, 2018;55(2):297–306.
  - This reference is of outstanding importance because it presents epidemiological data phenotyping neuropathic cancer pain (based on patient-reported outcome data) and modelling the predictive relationship between the prevalence of neuropathic cancer pain and exposure to anti-cancer therapies and receipt of analgesic therapies.
- Shkodra M, Mulvey M, Fallon M, Brunelli C, Zecca E, Bracchi P, Caputo M, Massa G, Lo Dico S, Rolke R, Kaasa S, Caraceni A. Application and accuracy of the EAPC/IASP diagnostic algorithm for neuropathic cancer pain and quantitative sensory testing profile in patients with pain due to cancer. Pain Rep. 2024;9(2):e1140.
  - This reference is of outstanding importance because it presented clinical trial data on the application of the EAPC diagnostic algorithm for neuropathic cancer pain, in additional to characterising the somatosensory profiles associated with altered sensory perception.
- Fallon MT. Neuropathic pain in cancer. Br J Anaesth. 2013;111(1):105–11.
  - This reference is of importance because it presents a clear and clinically relevant summary of the underlying pathophysiology of neuropathic pain in cancer, alongside a clear description of clinical management.
- Piano V, Schalkwijk A, Burgers J, Verhagen S, Kress H, Hekster Y, Lanteri-Minet M, Engels Y, Vissers K. Guidelines for neuropathic pain management in patients with cancer: a European survey and comparison. Pain Pract. 2013;13(5):349–57.
  - This reference is of importance because it developed an inventory of clinical practice guidelines for the treatment of neuropathic cancer pain in Europe. It reviewed and appraised the process of their development and suggested recommendations to improve the development of clinical practice guidelines.
- Johnson MI, Paley CA, Jones G, Mulvey MR, Wittkopf PG. Efficacy and safety of transcutaneous electrical nerve stimulation (TENS) for acute and chronic pain in adults: a systematic review and meta-analysis of 381 studies (the meta-TENS study). BMJ Open, 2022;12(2):e051073.
  - Of importance in that this review provides a wide-ranging systematic review and meta-analysis of TENS for all types of pain and against all comparators, finding moderate-level certainty of the efficacy of TENS either during or immediately after the application of TENS compared with placebo. TENS was shown to have a good safety profile.
- Ma X, Chen W, Yang NN, Wang L, Hao XW, Tan CX, Li HP, Liu CZ. Potential mechanisms of acupuncture for neuropathic pain based on somatosensory system. Front Neurosci. 2022;16:940343.
  - ○This reference is of importance as it explains in detail the evidence supporting a neurophysiological rationale for use of acupuncture for neuropathic pain, based on the somatosensory system and involving central and peripheral mechanisms.

## Data Availability

No datasets were generated or analysed during the current study.
